# In vitro toxicity and bioimaging studies of gold nanorods formulations coated with biofunctional thiol-PEG molecules and Pluronic block copolymers

**DOI:** 10.3762/bjnano.5.64

**Published:** 2014-04-30

**Authors:** Tianxun Gong, Douglas Goh, Malini Olivo, Ken-Tye Yong

**Affiliations:** 1 Bio-Optical Imaging Group, Singapore Bioimaging Consortium (SBIC), Agency for Science Technology and Research (A*STAR), 11 Biopolis Way, 138667 Singapore; 2 School of Electrical and Electronic Engineering, Nanyang Technological University, 639798 Singapore; 3 School of Physics, National University of Ireland, Galway, Ireland

**Keywords:** cancer cells, dark-field imaging, gold nanorods, PEG-SH, PEO–PPO–PEO

## Abstract

In this work, we investigated the cytotoxicity, colloidal stability and optical property of gold nanorods before and after functionalizing them with thiolated PEG and Pluronic triblock copolymer (PEO–PPO–PEO) molecules. The morphology of functionalized gold nanorods was characterized by UV–visible absorption spectroscopy, transmission electron microscopy, and dynamic light scattering. Solution phase synthesis of gold nanorods has remained the method of choice for obtaining varying shapes and aspect ratios of rod nanoparticles. This method typically involves the use of cetyltrimethylammonium bromide (CTAB) surfactants as directing agents to grow gold nanorods in the solution phase. The as-synthesized gold nanorods surfaces are terminated with CTAB molecules and this formulation gives rise to adverse toxicity in vitro and in vivo. To employ the gold nanorods for biological studies, it is important to eliminate or minimize the exposure of CTAB molecules from the gold nanorods surface to the local environment such as cells or tissues. Complete removal of CTAB molecules from the gold nanorods surface is unfeasible as this will render the gold nanorods structurally unstable, causing the aggregation of particles. Here, we investigate the individual use of thiolated PEG and PEO–PPO–PEO as capping agents to reduce the cytotoxicity of gold nanorods formulation, while maintaining the optical, colloidal, and structural properties of gold nanorods. We found that encapsulating gold nanorods with the thiolated PEG or PEO–PPO–PEO molecules guarantees the stability and biocompatibility of the nanoformulation. However, excessive use of these molecules during the passivation process leads to a reduction in the overall cell viability. We also demonstrate the use of the functionalized gold nanorods as scattering probes for dark-field imaging of cancer cells thereby demonstrating their biocompatibility. Our results offer a unique solution for the future development of safe scattering color probes for clinical applications such as the long term imaging of cells and tissues.

## Introduction

Gold nanorods (AuNRs) have been widely adopted for biological applications due to their unique plasmonic properties. One of the most important characteristics of AuNRs is that as light interacts with them, localized surface plasmon resonance (LSPR) is excited and locally oscillates around the particle [1]. LSPRs are electromagnetic modes associated with the collective oscillations of the free electrons confined to the nanoscale size. AuNRs have the unique ability to enhance the electromagnetic field within sub-wavelength regions adjacent to their surfaces under resonance excitation. The optical cross section of AuNRs is comparable to gold nanospheres and nanoshells, but the smaller effective dimension of AuNRs makes them useful for the targeted delivery into biological cells. AuNRs with larger aspect ratios and smaller effective radii are excellent photo-absorbing nanoparticles, while those with a larger effective radius have a higher scattering contrast signal [2]. These remarkable absorption and scattering capabilities make AuNRs promising candidates for bioimaging and biosensors [3–4].

AuNRs possess two SPR absorption peaks. One peak is located at the shorter wavelength (transverse plasmon peak) where light is transmitted across the transverse direction. The second peak can be found at the longer wavelength (longitudinal plasmon peak) where light is transmitted along the longitudinal direction [5]. The location of the longitudinal plasmon peak is highly dependent on the size, shape and aggregation state of the AuNRs. By carefully adjusting the length and diameter of AuNRs particle, one is able to manipulate their longitudinal absorption peak within the range from 600 to 1500 nm [6]. It is well recognized that near infrared (NIR) light is able to penetrate the human tissue up to a few centimeters since water and blood cells absorb light only minimally at this region. AuNRs can be designed to absorb light specifically in the NIR region so that heat is generated to damage cells and tissues. This property renders them useful for photothermal therapy and imaging of cancer [7–8]. In addition, the AuNRs surface can be functionalized with ligands for targeted drug delivery to support cancer therapy in vitro and in vivo [9]*.* Furthermore, it is well reported that AuNRs are often used for surface enhanced Raman spectroscopy (SERS) biosensing applications. This is based on the observation that a gold rod-like particle has a higher electric field at both ends of the rod [10–11] where it is particularly useful for enhancing the signals from Raman tags.

Over the past few years, the seed-mediated growth method proposed by Murphy and El-Sayed’s group has been commonly used for synthesizing AuNRs formulations [6,12–13]. Cetyltrimethylammonium bromide (CTAB) molecules are used as structure directing agents to support the formation of gold rod-like particles in the aqueous medium. The issue with CTAB, however, is that it forms a tightly bound cationic bilayer on the surface of the AuNR with the cationic trimethylammonium head group exposed to the external environment. The presence of CTAB on the AuNRs surface poses a threat to many biological systems as they are toxic to cells and tissues. As a result, CTAB-coated AuNRs are not suitable to be used for biomedical applications [14–15]. CTAB can be partially removed from the AuNRs surface by centrifugation, but the majority of the CTAB molecules remains on the particle surface and continues to exhibit toxicity to cells. On the other hand, repeated centrifugations results in structurally unstable AuNRs and causes them to aggregate and precipitate in solution. Also, CTAB-coated AuNRs are not suitable for in vitro and in vivo applications because they do not allow antibodies or antigens to be linked to their surface for targeted delivery and imaging [16–17]. More importantly, one is not able to use CTAB-coated AuNRs as a carrier for drug delivery of water insoluble anti-cancer agents (e.g., doxorubicin, paclitaxel) to the cancerous area since their surface is hydrophilic and positively charge [18–19]. Therefore, a surface functionalization platform is needed to furnish a AuNR surface with a biocompatible polymer-coating for reducing their cytotoxicity while maintaining colloidal stability and allowing them to be conjugated for biomedical applications. Bio functional thiol-poly(ethylene glycol) (PEG-SH) molecules and Pluronic block copolymers (PEO–PPO–PEO) (see chemical formula of PEG-SH and Pluronic (PEO–PPO–PEO) in Supporting Information File 1, Figure S1) are commonly used to prepare non-ionic polymer encapsulated AuNRs with a stealth property for in vivo studies [20–23]. It is noteworthy that these PEG polymers can even be modified with additional functional groups such as a carboxyl and an amino group for the conjugation of targeting ligands_._ It is known that the CTAB bilayers on a AuNRs surface can be removed and replaced with PEG-SH molecules by means of the chemisorption process between the thiol moiety and the gold particle surface [24–25]. Pluronic is a commercially available triblock copolymer with a hydrophobic segment of poly(propylene oxide) (PPO) polymer sandwiched between two hydrophilic segments of PEO. In our previous study, we found that the hydrophobic PPO segment from the Pluronic block copolymer is able to bind to the hydrophobic part of CTAB molecules on AuNRs and form stable CTAB-polymer complexes [26–27].

Upon functionalizing AuNRs with either PEG-SH or PEO–PPO–PEO molecules, many physicochemical property of a gold nanoparticles formulation will be affected and this may impact their applications in sensing, imaging and targeted delivery. Thus, it is essential for the nanoparticle community to understand the effects of functionalizing PEG-SH or PEO–PPO–PEO molecules on the AuNRs surface and their corresponding impact on biological systems. In this work, we systematically study the cytotoxicity, colloidal stability, and optical property of AuNRs before and after functionalizing them with PEG-SH and PEO–PPO–PEO molecules. These AuNRs formulations were characterized by using UV–vis spectroscopy, transmission electron microscopy (TEM), cell viability assay, dynamic light scattering (DLS), and dark-field imaging microscopy. The non-specific uptake of these AuNRs by cells was also studied under dark-field microscopy. Our work demonstrates that the coating of AuNRs surfaces with PEG-SH or PEO–PPO–PEO molecules significantly improved the colloidal and optical stability of the gold nanoformulation. No aggregation is found even a few weeks after the preparation. More importantly, the cell viability and dark-field imaging studies indicate that the AuNRs functionalized with PEG-SH or PEO–PPO–PEO molecules have minimal cytotoxicity and they can be used for long term in vitro and in vivo imaging study.

## Experimental

**Materials:** Hydrogen tetrachloroaurate(III) trihyrate (HAuCl_4_·3H_2_O), cetylmethylammonium bromide (CTAB), sodium borohydride (NaBH_4_), silver nitrate (AgNO_3_), L-ascorbic acid, trisodium citrate (Na_3_C_6_H_5_O_7_), Pluronic F127, and the cell counting kit (CCK8) were purchased from Sigma-Aldrich. PEG-SH (CH_3_O–PEG-SH) was purchased from Rapp Polymere. Dulbecco’s modified Eagles’s medium (DMEM) and 1× phosphate buffer sulphate (PBS) were prepared in-house. Fetal bovine serum (FBS) and penicillin/streptomycin (Pen Strep) were purchased from Gibco^®^. The clean-mount solution for fixing a glass cover slip over 8-chamber slides was purchased from electron microscopy sciences.

**AuNRs synthesis and characterization:** Synthesis of AuNRs was adapted from Nikhoobakt et al. [6]. As described in [27], 5 mL of 0.5 mM HAuCl_4_ was added to 5 mL of 200 mM CTAB to obtain an amber colored solution. 600 μL of 10 mM NaBH_4_ was then added to the solution and stirred vigorously for a minute. A light brown seed solution was obtained. AuNRs were synthesized by a seed-mediated method, and 5 mL of 1 mM HAuCl_4_ was added to 5 mL of 200 mM CTAB and stirred. 350 μL of 4 mM AgNO_3_ was then added. 70 μL of 78.8 mM of L-ascorbic acid was added, and a colorless solution was formed. 18 µL of the seed solution was injected into the growth solution and left to form AuNRs for an hour at room temperature. The AuNRs solution was centrifuged at 10,000 rpm for 10 min and suspended in water. This washing step was repeated 3 times to remove excess CTAB. For AuNRs encapsulation, after three centrifugations, the supernatant were taken out and the AuNRs pellet was left in the centrifuge tube without suspending them in water. A transmission electron microscope (TEM) was used with JEOL JEM-1010 to characterize the shapes and sizes of the AuNRs. The TEM specimens were prepared on 200 mesh nickel-coated grids. UV–vis absorption spectra of AuNRs were obtained by using a Hitachi U-2900 with a double-beam optical system and a spectral bandpass of 1.5 nm over the spectrophotometric with a wavelength range of 400 to 1100 mm. The specimen was placed in a quartz cuvette for measurement and deionized water was used as a reference.

**Functionalization of AuNRs with PEG-SH or Pluronic molecules:** In a similar way as described in [27], 1 mL of Pluronic F127 or PEG-SH solution of various concentrations (10 nM, 100 nM, 1 µM, 10 µM, 100 µM and 1 mM) was added to the AuNRs pellet. The resultant solutions were left to be incubated for 1 h and then centrifuged once to remove excess Pluronic or PEG-SH solution. The functionalized AuNRs were then resuspended in water. Concentrations of the AuNRs solutions were fixed at an optical density of 1.5 for our studies.

**Cell culture and cell viability:** As described in [27], oral squamous cell carcinoma (OSCC) cell line was cultured in DMEM containing 10% FBS with Pen Strep. All cultures were kept at 37 °C with 5% CO_2_. 5,000 cells were seeded in a 96-well plate for 24 h before loading each well with 10 µL of AuNRs solution (concentrations of all the solutions were fixed at an optical density of 1.5 with a UV–vis spectrophotometer). After a further incubation of the cells for 24 h, 10 µL of CCK8 was added to each well followed by another incubation of 4 h in the dark at 37 °C with 5% (v/v) CO_2_. Cell population absorbance was performed with the SpectraMax 384 Plus spectral analyzer. The absorbance from the tetrazolium dye in CCK8 was measured at 450 nm excitation.

**In vitro dark-field imaging study:** As described in [27], 5,000 cells suspended in media were seeded in each well of the 8-well chamber glass slide and allowed to be confluent. Media was then removed and the slide was rinsed with PBS. Media was replenished in the wells. The corresponding synthesized substances were loaded and allowed to incubate for four hours at room temperature and pressure in the dark. The media and synthesized substances were removed and rinsed with PBS again, and the cells were fixed with 4% paraformaldehyde for 10 min. Thereafter, 4% paraformaldehyde was removed and rinsed with PBS. The well was removed and a cover slip was fastened with a layer of clean-mount on the slide. Dark-field imaging was performed with a Nikon Eclipse 80i at 100× magnification.

## Results and Discussion

**Figure 1:**
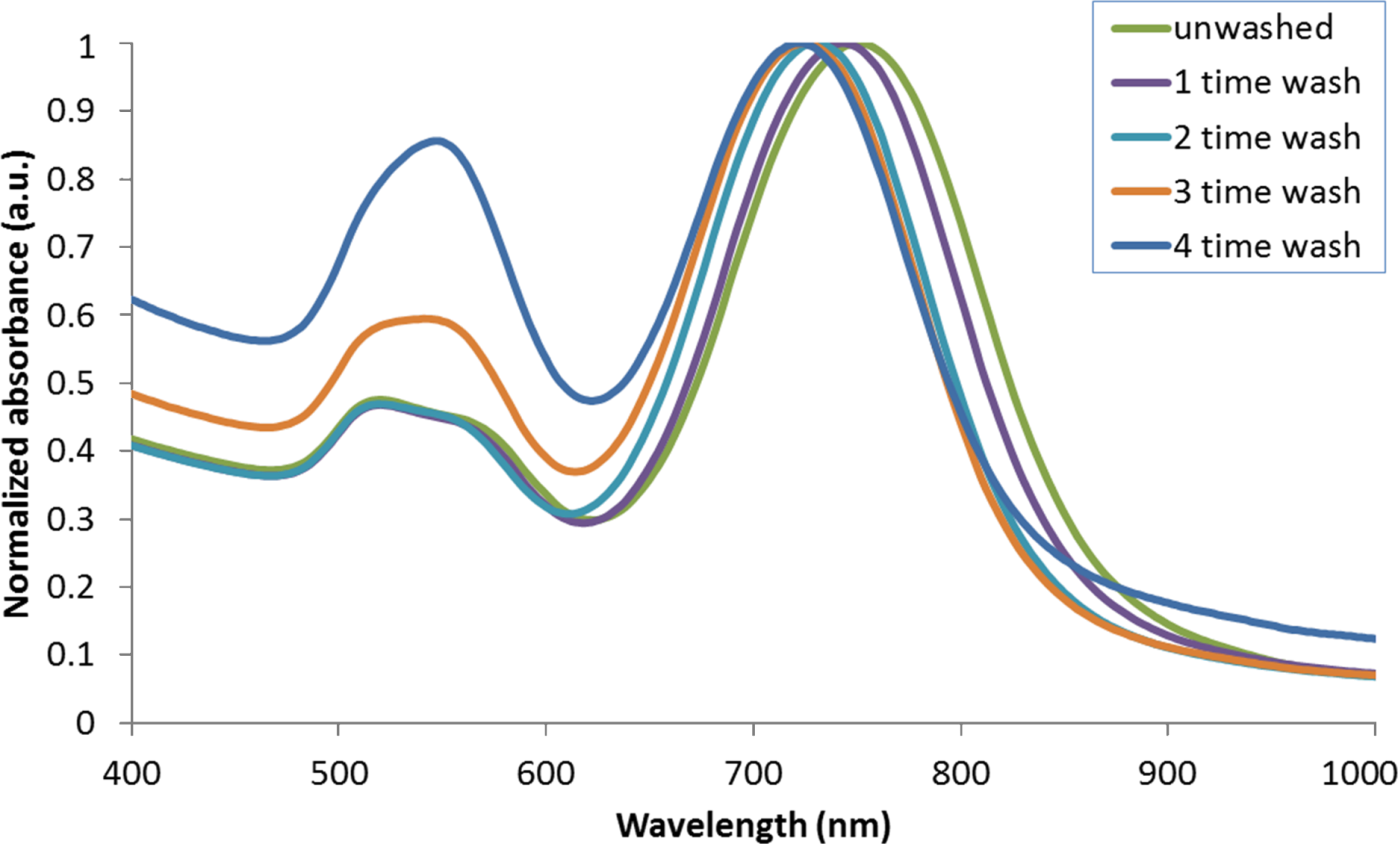
Normalized UV–vis absorption spectra of as-synthesized AuNRs and AuNRs washed one, two , three and four times by centrifugation.

**Figure 2:**
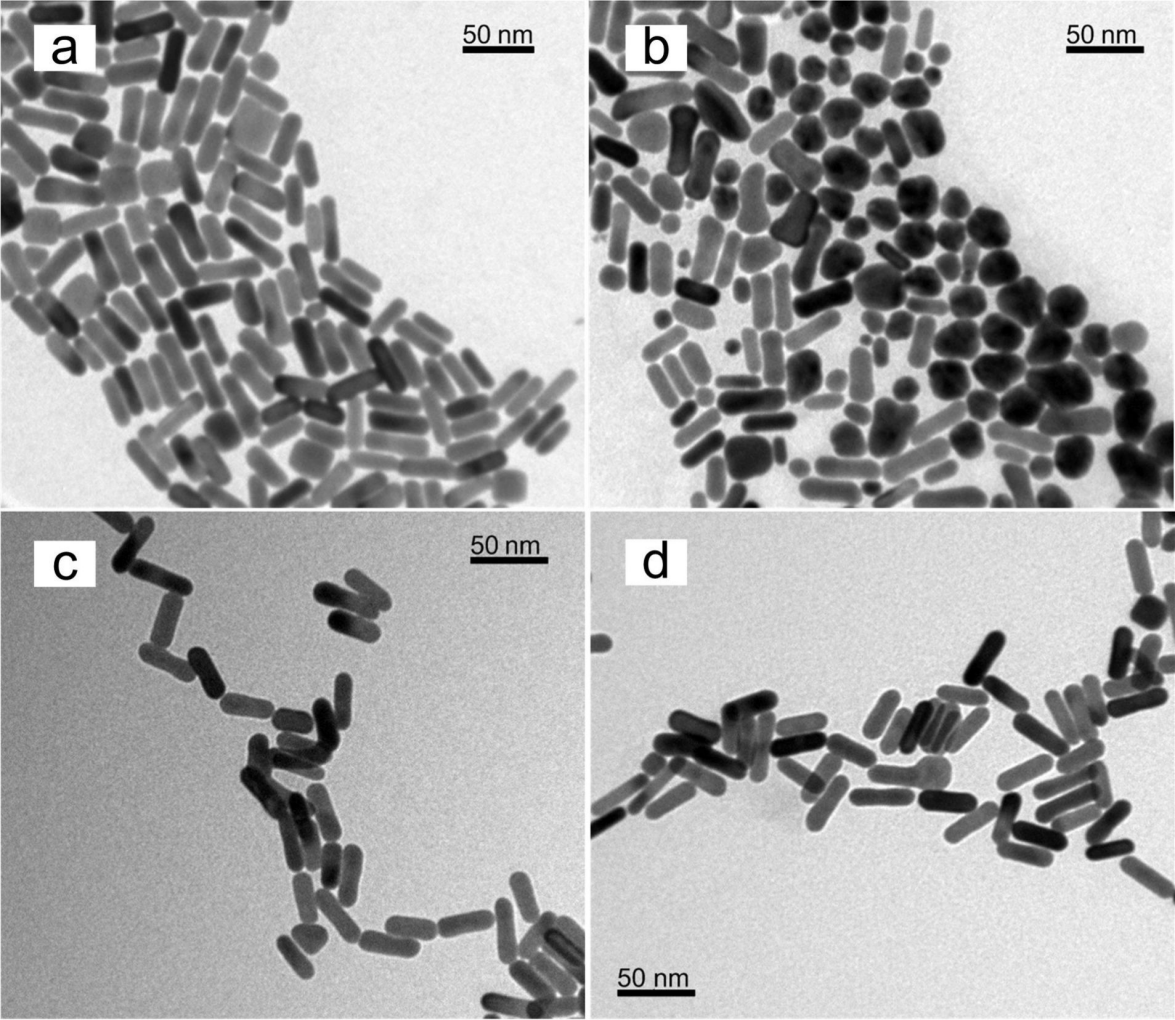
TEM images of (a) as-synthesized AuNRs, (b) AuNRs washed 4 times by centrifugation, (c) PEGylated AuNRs and (d) PEO–PPO–PEO encapsulated AuNRs.

We used a seed-mediated approach to synthesize AuNR particles with a longitudinal SPR at 750 nm. During the formation of the AuNRs, the head group of CTAB molecules preferentially binds to specific crystallographic faces of gold. Thus the gold atoms are directed to deposit on selective faces of gold and attain anisotropic nanoparticles in the solution medium [6,28–29]. In this process, CTAB forms a tightly bound cationic bilayer on the surface of AuNRs and CTAB-coated particles are known to be cytotoxic. We found that a major fraction of the CTAB molecules can be removed from the gold particle surface by multiple centrifugation steps. However, the washing steps affect the stability of the AuNRs and cause them to aggregate into precipitates in the solution. CTAB molecules are known to serve as surfactants for passivating the particles surface and maintaining the colloidal stability of the particles. The disruption and removal of CTAB from the gold particle surface results in large attractive interparticle forces, so that the particles form aggregates. To better understand this process UV–vis absorption spectra were measured for AuNR formulations before and after different treatments with washing steps. Figure 1 shows the normalized absorption spectra of AuNRs at different stages of washing treatments. A comparison of the absorption spectrum of as-synthesized AuNRs and treated AuNRs revealed that every round of washing caused a slight blue shift of the longitudinal SPR peak and an increase in the intensity of the transverse SPR peak starting from the third round of washing treatment. As the longitudinal SPR peak is determined by the aspect ratio of the AuNRs, the blue shift of this peak indicates that the aspect ratio of the AuNRs decreases as nanoparticles start to form large aggregates in the solution. To determine the morphology of AuNR particles at various stages, we performed TEM analysis on as-synthesized AuNRs and AuNRs after four rounds of washing treatment. The TEM image in Figure 2a shows that more than 90% of the as-synthesized AuNR particles are rod-like in dimension. Figure 2b indicates that a large fraction of AuNRs formed larger spherical aggregates, accounting for less than 50% rod-like particles.

To improve the compatibility of AuNRs for biological applications, we functionalized the particles with PEG-SH or PEO–PPO–PEO molecules. Hydrophobic PPO chains of Pluronic copolymer are able to bind to the hydrophobic tails of CTAB molecules, so that PEO–PPO–PEO molecules on the surface of CTAB-coated AuNRs are passivated. The hydrophilic PEO chains of the copolymer are then favorably interacting with the aqueous phase thereby maintaining the overall colloidal solubility of the AuNRs passivated by the Pluronic copolymer. Pluronic F127 was chosen as it has PPO and PEO chains of comparable length. Therefore, the advantages of these two block copolymers are combined to provide a better surface passivation on the particles and a better colloidal stability [30–32]. PEG-SH and Pluronic encapsulated AuNRs were prepared by retrieving particles after the third round of the washing treatment. Figure 2c and Figure 2d show the TEM images of AuNRs functionalized with PEG-SH and PEO–PPO–PEO molecules, respectively. In comparison to Figure 2a, the overall size and shape of AuNRs functionalized with either PEG-SH or PEO–PPO–PEO molecules remain the same, thus demonstrating that these two polymer molecules are suitable to be utilized in engineering the particle surface in way that maintains the optical and colloidal stability of AuNRs.

**Figure 3:**
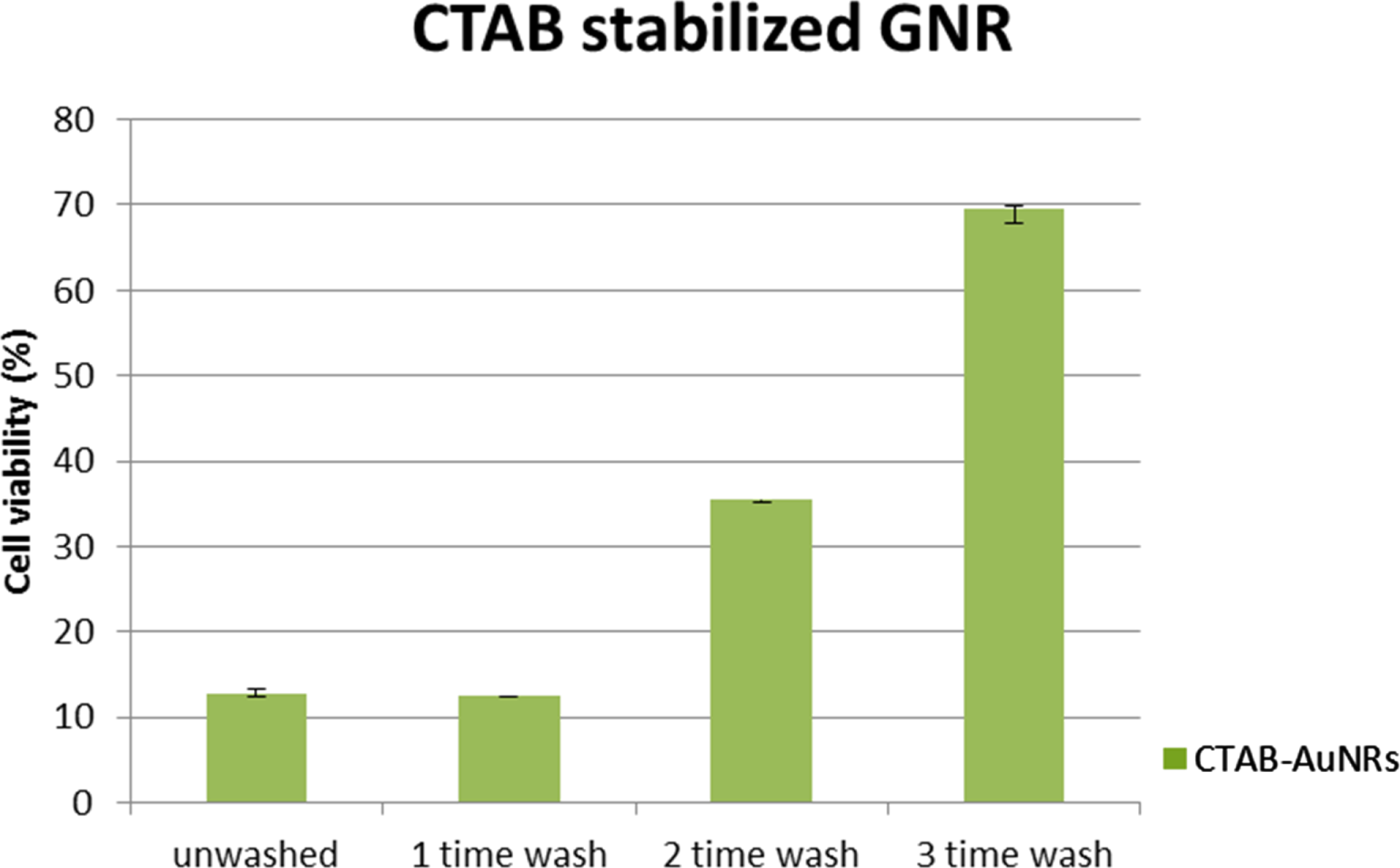
Relative cell viability of OSCC cells 24 h post-treatment. The cells were treated with as-synthesized AuNRs and AuNRs after different rounds of washing treatments.

**Figure 4:**
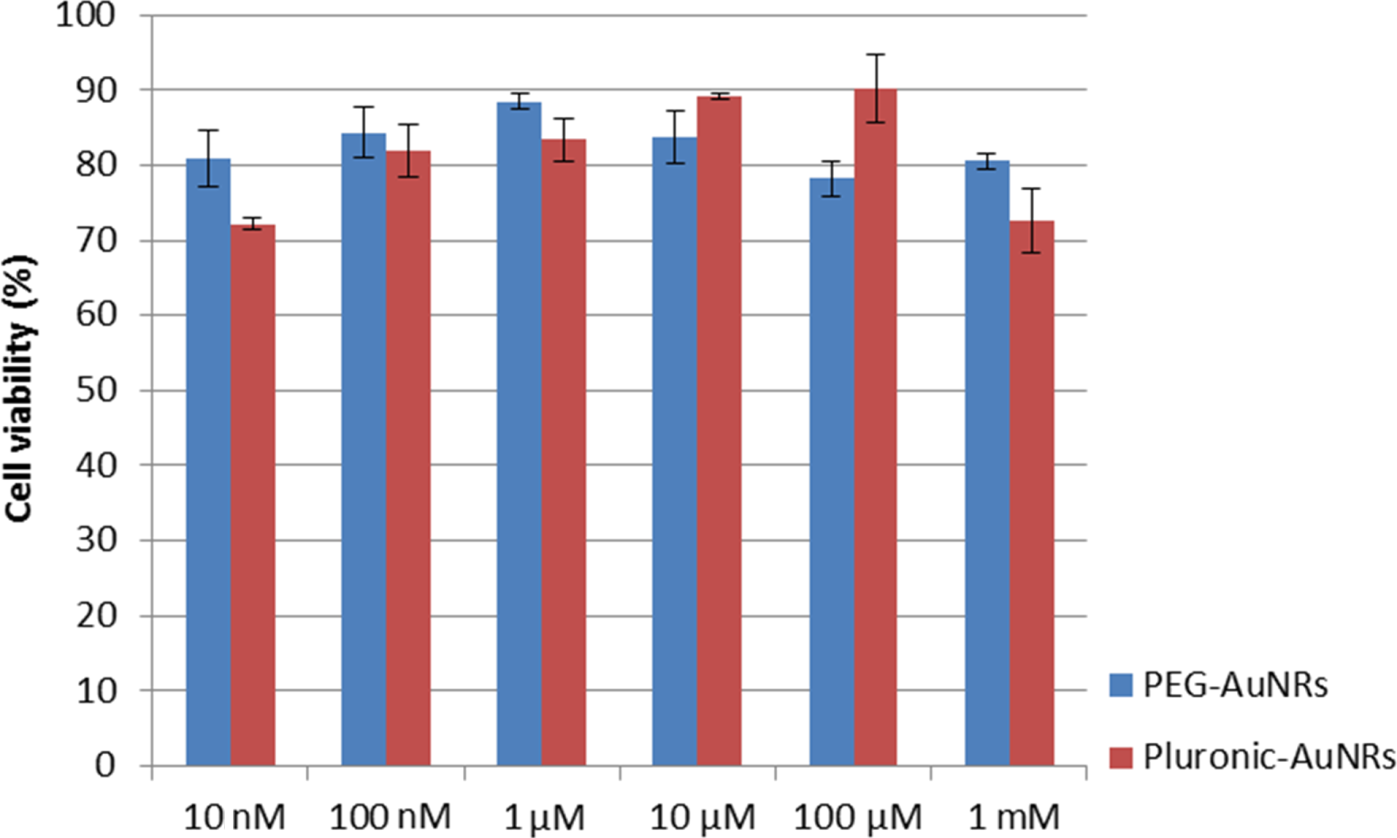
Cell viability of AuNRs encapsulated with different concentrations of PEG-SH or Pluronic triblock copolymer. The concentration of PEG-SH and Pluronic adopted for encapsulation ranges from 10 nM to 1 mM.

To systematically study the cytotoxicity of these functionalized AuNRs formulations, different concentrations of PEG-SH and PEO–PPO–PEO were used to react with CTAB-coated AuNRs and thereby producing AuNR formulations with a different surface coverage of PEG-SH and PEO–PPO–PEO molecules. The cytotoxicity between the as-synthesized AuNRs and AuNRs after varying times of washing treatment was also compared. As shown in Figure 3, the as-synthesized AuNR formulation has excess CTAB molecules not only on the particle surface but also in the solution. This formulation exhibits a high toxicity to the cells. The cell viability of OSCC cells is maintained around 10% at 24 h post-treatment. Upon treating the OSCC cells with AuNRs after three rounds of washing, we were able to observe a significant increase in the cell viability to up to 70%. This demonstrates that the toxicity of the formulation was drastically reduced by removing CTAB surfactants from the particle suspension. However, further washing treatments to the AuNRs is infeasible because the particles will become unstable in the absence of CTAB surfactants in the suspension. The cell viability of AuNRs functionalized by different concentrations of PEG-SH or PEO–PPO–PEO surfactants is shown in Figure 4. It is clear that functionalizing the AuNRs surface with PEG-SH and PEO–PPO–PEO molecules can dramatically reduce the cytotoxicity of the formulation. This can be observed from the cell viability assay where the percentage is maintained at nearly 90% with concentrations of PEG-SH and PEO–PPO–PEO ranging from 10 nm to 1 mM. However, we observed that there is a concentration of PEG-SH and PEO–PPO–PEO molecules used for synthesizing AuNRs which yields the lowest cytotoxicity. We found that the use of 1 µM PEG-SH or 100 µM PEO–PPO–PEO to treat CTAB-coated AuNRs is able to produce a highly biocompatible particles formulation for in vitro applications. The reaction of CTAB-coated AuNRs with higher concentrations of PEG-SH and PEO–PPO–PEO molecules resulted in decreased cell viabilities. This may be caused by the impact of forming a thicker coating layer on the AuNRs surface [33]. Many groups have reported on the encapsulation of AuNRs with other polymer coatings to improve the biocompatibility of the rod nanoparticle formulations. For example, Alkilany et al. demonstrated the use of polyacrylic acid (PAA) and polyelectrolyte poly(allylamine) hydrochloride (PAH) to coat AuNRs surface. The coating was performed on as-synthesized CTAB-coated AuNRs after a washing treatment by centrifugation [15]. The CTAB-coated AuNRs solution was found to reduce cell viability by 30%, while both PAA-coated AuNRs and PAH-PAA-coated AuNRs were found to be non-toxic with a cell viability of about 90%. It was also found that the effective hydrodynamic diameter of PAA-coated AuNRs and PAH-PAA-coated AuNRs increases from 20 nm (CTAB-coated AuNRs) to 25 nm and 30 nm, respectively, indicating the successful coating of a polymer layer on the AuNRs surface. Wang et al. also reported the cytotoxicity of AuNRs under different conditions [34]. They found that the as-synthesized AuNRs formulation was highly toxic and a very low cellviability result (≈10%) was observed for this formulation. However, after 3 washing treatments by centrifugation, the cytotoxicity of CTAB-coated AuNRs was found to decrease, even though not to the extent which allows their usage for biological studies. Similar observations were made in our study.

**Figure 5:**
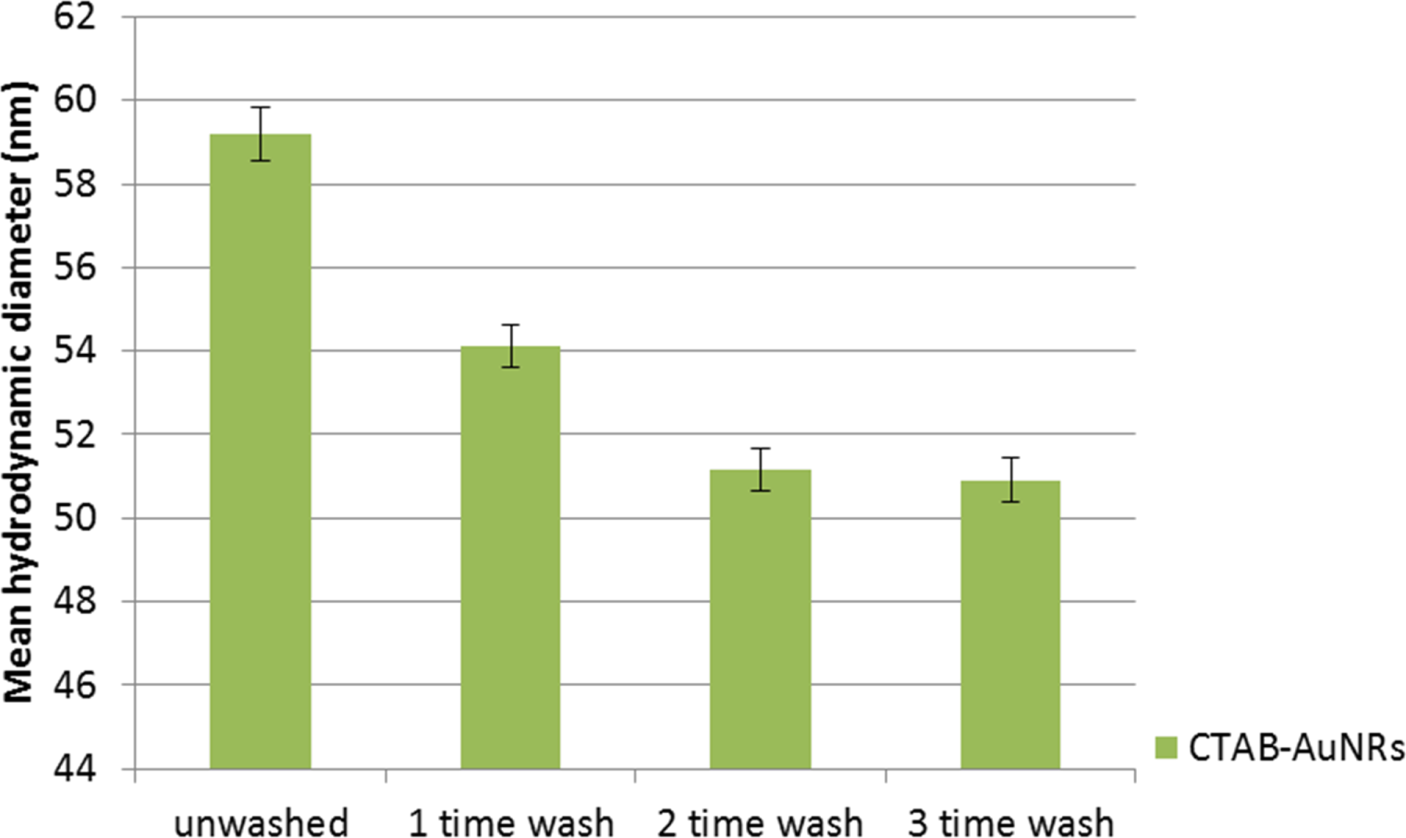
Mean hydrodynamic diameters of as-synthesized AuNRs (unwashed) and AuNRs washed three times by centrifugation. The hydrodynamic diameter is inferred from the diameter of the outermost encapsulation layer on a particle.

**Figure 6:**
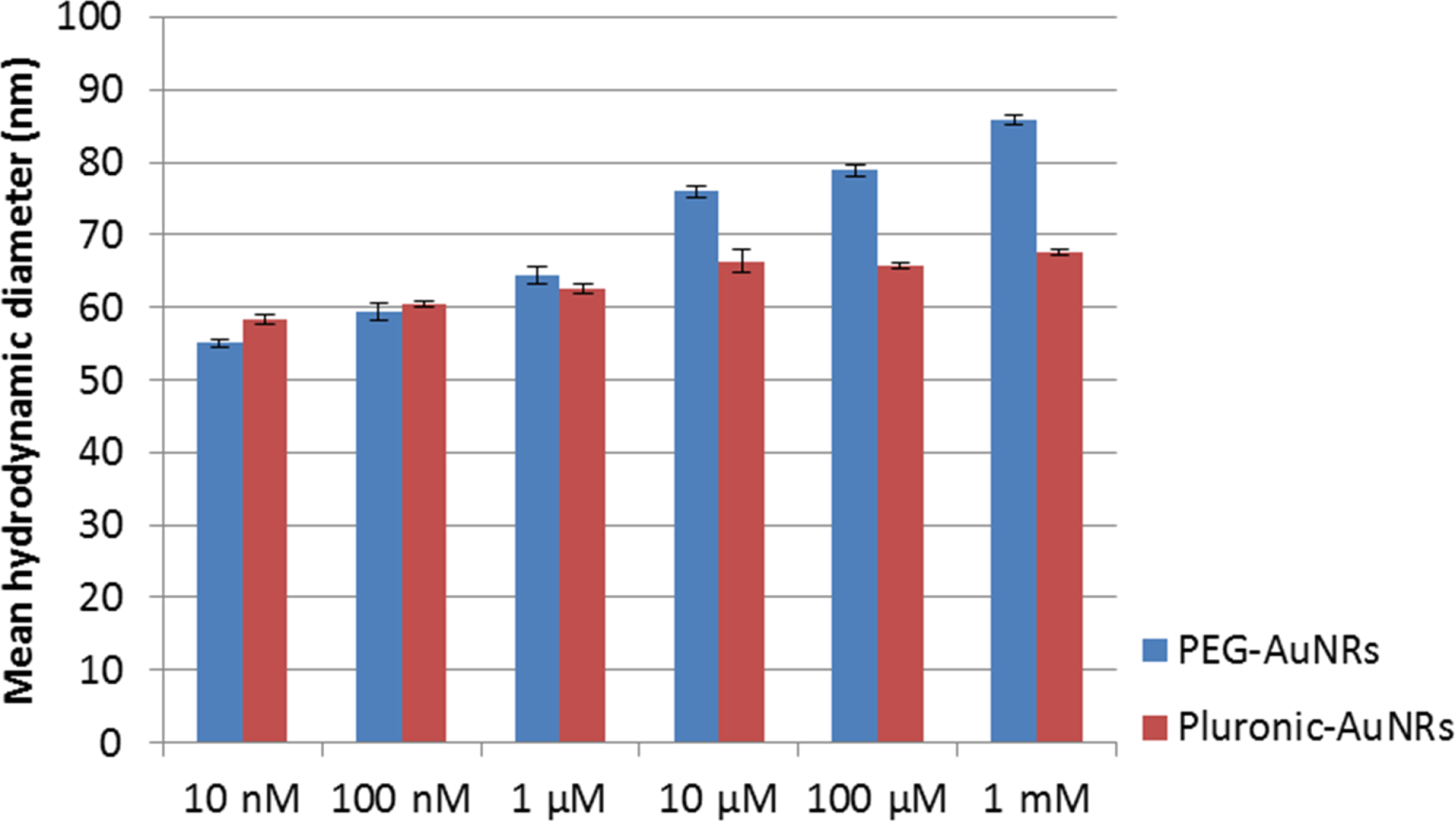
Mean hydrodynamic diameter of AuNRs encapsulated with different concentrations of PEG-SH or Pluronic.

DLS experiments were performed in our study to determine the hydrodynamic diameter and the colloidal stability of the prepared AuNR formulations. Figure 5 shows the mean hydrodynamic diameter of CTAB-coated AuNRs before and after different rounds of washing treatment. A slight decrease (6 to 7 nm) in the overall hydrodynamic diameter of the nanoparticles formulation was observed after an additional round of washing. This indicates that a fraction of CTAB molecules was removed from the surface of the AuNRs with every round of washing by centrifugation. In general, we observed that AuNRs became less stable in the aqueous phase and formed aggregates after two to three rounds of washing. We observed that the mean hydrodynamic diameter of AuNRs increases slightly when the concentration of PEO–PPO–PEO and PEG-SH in the reaction mixture is increased (Figure 6). According to TEM analysis the hydrodynamic sizes of the AuNRs functionalized with PEG-SH or PEO–PPO–PEO are found to be larger than those of AuNRs. This is mainly due to the solvated polymer layers of PEO–PPO–PEO or PEG-SH on the AuNRs surface. In our study, only AuNRs which underwent three washing treatments were used for functionalization with polymer molecules. The hydrodynamic diameter of the AuNRs was determined to be 55 and 58 nm after passivating their surface with PEO–PPO–PEO and PEG-SH molecules.

**Figure 7:**
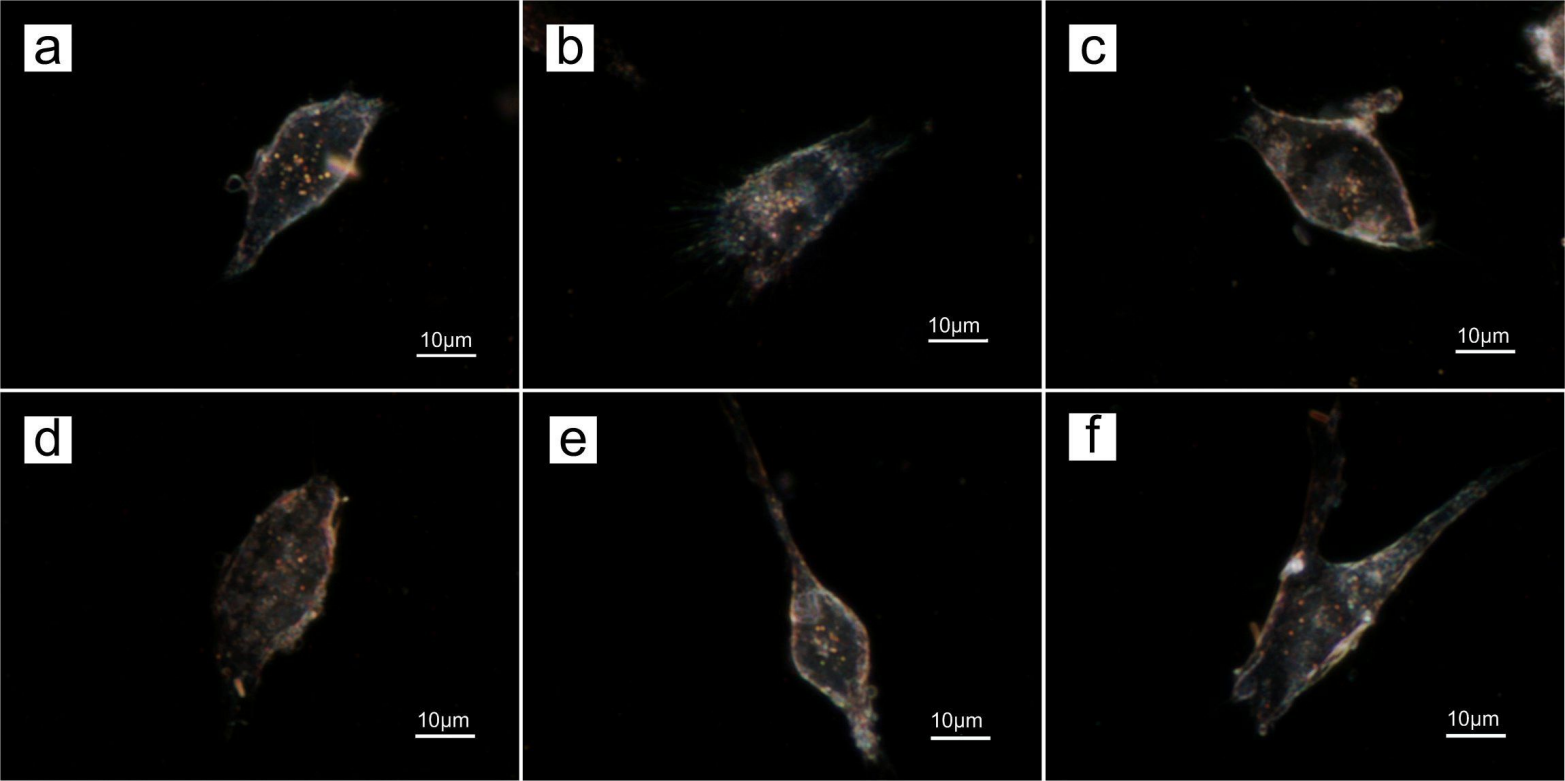
Dark-field images of OSCC cells with AuNRs encapsulated with (a) 10 nM, (b) 1 µM, (c) 1 mM of PEG-SH and (d) 10 nM, (e) 100 µM (f) 1 mM of Pluronic F127.

For in vitro imaging study, OSCC cells were treated with AuNRs functionalized with either PEG-SH or PEO–PPO–PEO molecules for evaluating their biocompatibility. In accordance with the cell viability results in Figure 4, three concentrations of PEG-SH (10 nM, 1 µM, 1 mM) and Pluronic (10 nM, 100 µM, 1 mM) were used to passivate AuNRs, and these formulations were employed for in vitro dark-field imaging. Figure 7 shows dark-field images of OSCC cells with the encapsulated AuNRs synthesized in this study. The bright red and orange scattered spots located within the cells suggest that the AuNRs were internalized into the cells by non-specific cellular uptake since no biomolecules were attached to our gold formulations. Huang et al. performed a similar experiment where AuNRs were also observed to be internalized by malignant oral epithelial cell lines and the extinction spectra analysis confirmed that the scattering colors within the cells was caused by nanoparticles [7]. Thus, the coupling of the inherent scattering property of AuNRs with the use of polymer-based encapsulation further facilitates the use of AuNRs as biocompatible in vivo probes.

## Conclusion

In this work, we studied the cytotoxicity, colloidal stability and optical property of AuNRs before and after functionalizing them with PEG-SH and PEO–PPO–PEO molecules. The as-synthesized AuNR surfaces are functionalized with CTAB molecules. This formulation is highly toxic and not suitable to be used for any biological applications. To employ the AuNRs for biological studies, the surface of AuNRs needs to be passivated with a biocompatible polymer coating. The encapsulation of AuNRs with PEG-SH or PEO–PPO–PEO molecules produces biocompatible AuNRs formulations. These formulations lead to stable colloidal solutions and can be readily used for dark-field imaging of cancer cells. We believe that this work provides useful insight for developing new protocols for preparing biocompatible AuNRs for applications ranging from cell imaging to targeted in vivo drug delivery.

## Supporting Information

File 1 Chemical formula of PEG-SH and Pluronic (PEO–PPO–PEO).
