# Supplementary material for: In vitro toxicity and bioimaging studies of gold nanorods formulations coated with biofunctional thiol-PEG molecules and Pluronic block copolymers
Source: Beilstein J Nanotechnol. 2014 Apr 30;5:546–53. doi: 10.3762/bjnano.5.64 (PMC4077316; doi:10.3762/bjnano.5.64)
Supplement: File 1 — Chemical formula of PEG-SH and Pluronic (PEO–PPO–PEO). [file Beilstein_J_Nanotechnol-05-546-s001.pdf]

Supporting Information

for

In vitro toxicity and bioimaging studies of gold nanorods formulations coated with biofunctional thiol-PEG molecules and Pluronic block copolymers

Tianxun Gong[1,2], Douglas Goh[1], Malini Olivo[1,3] and Ken-Tye Yong*[2]

Address: [1]Bio-Optical Imaging Group, Singapore Bioimaging Consortium (SBIC), Agency for Science Technology and Research (A*STAR), 11 Biopolis Way, 138667 Singapore, [2]School of Electrical and Electronic Engineering, Nanyang Technological University, 639798 Singapore and [3]School of Physics, National University of Ireland, Galway, Ireland

Email: Ken-Tye Yong - ktyong@ntu.edu.sg

*Corresponding author

Chemical formula of PEG-SH and Pluronic (PEO–PPO–PEO)

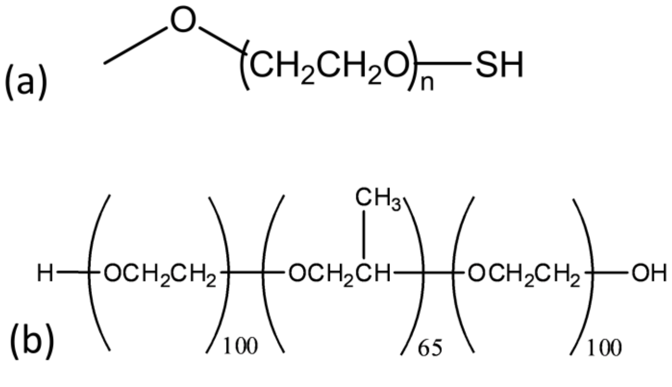

Figure S1: Chemical formula of (a) PEG-SH and (b) Pluronic F127.
